# Comprehensive Gene Expression Analysis in Papillary Thyroid Carcinoma Reveals a Transcriptional Profile Associated with Reduced Radioiodine Avidity

**DOI:** 10.1007/s12022-025-09849-0

**Published:** 2025-02-21

**Authors:** Vincenzo Condello, Carlotta Marchettini, Catharina Ihre-Lundgren, Joachim N. Nilsson, C. Christofer Juhlin

**Affiliations:** 1 Department of Oncology-Pathology, Karolinska Institutet, Stockholm, Sweden; 2 Department of Molecular Medicine and Surgery, Karolinska Institutet, Stockholm, Sweden; 3 Department of Breast, Endocrine Tumors, and Sarcoma, Karolinska University Hospital, Stockholm, Sweden; 4 Department of Nuclear Medicine and Medical Physics, Karolinska University Hospital, Stockholm, Sweden; 5 Department of Pathology and Cancer Diagnostics, Karolinska University Hospital, Stockholm, Sweden

**Keywords:** Radioiodine avidity, Papillary thyroid carcinoma, RNA-sequencing, *ANO1*

## Abstract

Papillary thyroid carcinoma (PTC) is the most common form of well-differentiated thyroid cancer (WDTC) and generally has a favorable prognosis. However, subsets of these tumors can metastasize, leading to aggressive disease progression and poorer clinical outcomes. Radioactive iodine (RAI) therapy is routinely given in the adjuvant setting following thyroidectomy and lymph node dissection for WDTC. Nevertheless, its therapeutic efficacy is limited to tumors with high iodine avidity. Early post-surgical classification of thyroid cancers as either iodine-avid or refractory is crucial for enabling more personalized and effective treatment strategies. In this study, we aimed to identify transcriptomic determinants associated with RAI refractoriness (RAI-R) to improve prognostication. We collected clinicopathologic data and conducted RNA-seq on 36 tissue samples (18 high-avidity and 18 low-avidity), each uniquely characterized by ex vivo iodine concentration measurements taken directly from surgical specimens. Whole-transcriptomic analysis identified 63 differentially expressed genes, with six (*S100A4*, *CRTC2*, *ANO1*, *WWTR1*, *DEPTOR*, *MT1G*) showing consistent deregulation. The expression of ANO1, an established iodine transporter at the apical membrane of the thyroid follicular cells, correlated significantly with iodine avidity (*r* = 0.54). Validation via RT-qPCR confirmed differential expression trends. Gene ontology and pathway enrichment analyses highlighted thyroid hormone synthesis, PI3K-AKT, and MAPK signaling pathways as key regulators of RAI avidity. A refined multivariate predictive model incorporating *ANO1* mRNA expression, histological subtypes, and sample type demonstrated strong predictive performance (adjusted *R*^2^ = 0.55). These findings suggest *ANO1* as a promising biomarker for predicting iodine avidity in thyroid cancer.

**Supplementary Information:**

The online version contains supplementary material available at 10.1007/s12022-025-09849-0.

## Introduction

Cancers arising from thyroid follicular cells are typically straightforward to categorize based on their histological characteristics. Most cases are well-differentiated tumors (WDTC), which are generally indolent, with an overall good prognosis, and are typically managed with minimally invasive therapies [1]. Among these, papillary thyroid carcinoma (PTC) is the most common type of WDTC [2].

Radioactive iodine (RAI) therapy is a first-line treatment commonly used in managing patients with WDTC. This approach significantly enhances survival and disease control in high-risk patients while enabling efficient follow-up of low-risk cases. However, its therapeutic efficacy is limited to tumors that exhibit high iodine avidity [3]. Up to 15% of patients diagnosed with WDTC and approximately 50% of those with metastatic disease exhibit very low or no iodine avidity. This manifests as low or no uptake signal on RAI scans and lack of treatment response, signifying RAI refractory (RAI-R) disease [4]. RAI-R metastatic disease is associated with poor outcomes, with 5-year disease-specific survival rates ranging from 60 to 70%, and 10-year survival rates dropping as low as 10% [5]. Late identification of patients with RAI-R disease can lead to unnecessary treatments, delaying other potentially more effective therapies and increasing the risk of adverse side effects. The clinical significance of identifying RAI-R disease is further emphasized by recommendations to implement reflex molecular testing in thyroid carcinoma specimens where RAI-R disease is anticipated [6].

In recent years, it has been demonstrated that patient age, histological subtype, and mutations in *BRAF* and *TERT* promoter are reliable predictive factors for tumor response to RAI therapy [7–10]. While *BRAF* p.V600E mutations alone are not sufficient to drive thyroid carcinoma into a RAI-R state, their association with additional progression events, such as *TERT* promoter mutations, can lead to reduced iodine uptake. The RAI-R phenotype is not always associated with histological dedifferentiation, however, is commonly linked to molecular changes in the tumor, particularly reduced sodium-iodide symporter (NIS) expression [11, 12]. Loss-of-function mutations in the *SLC5A5* gene, which encodes the NIS protein, are rare in thyroid cancer. Instead, the impaired iodine transport typically results from silencing or post-translational modifications that hinder NIS localization to the plasma membrane [13, 14]. Constitutive activation of the mitogen-activated protein kinase (MAPK) pathway, driven by mutations or fusions in genes such as *RET*, *NTRK1*, and *BRAF*, seems to disrupt this regulation [15].

Recently, several studies have demonstrated that MAPK inhibition restores the expression of thyroid differentiation genes and enhances RAI avidity [16]. Targeted therapies, particularly MEK inhibitors, have proven effective in resensitizing subsets of RAI-R tumors, with success rates exceeding 50% [17]. Restoration of iodine avidity has also been reported after treatment with NTRK inhibitors in patients with *EML4::NTRK3* and *RET* gene fusions [18, 19]. Preclinical studies in transgenic mice driven by *STRN::ALK* gene fusion have also demonstrated that ALK fusion-induced NIS downregulation contributes to RAI-R by enhancing MAPK, JAK/STAT3, and PI3K/AKT/mTOR signaling pathways. Notably, ALK inhibitors have been shown to reverse this downregulation, offering a potential therapeutic strategy [20].

Individuals with RAI-R disease are often identified at an advanced stage, after undergoing multiple ineffective treatment cycles. Similarly, smaller but more aggressive tumors with moderate-to-low avidity that may benefit from higher doses of RAI to compensate for lower uptake are rarely identified at an early stage. Despite these insights, the factors influencing response or resistance to RAI remain elusive or not fully understood. In recent years, various efforts have aimed to address this issue; however, none have proposed new markers to predict refractoriness [21, 22]. This underscores the urgent need for better strategies to predict RAI-R at earlier stages.

In consideration of these factors, the present study sought to investigate the potential correlation between low iodine avidity and alterations in gene expression. The objectives encompassed identifying specific molecular and clinical factors associated with this phenotype, as well as evaluating their prognostic and therapeutic potential. Due to the rarity of tissue biopsies from clinically confirmed RAI-R thyroid carcinoma samples, we opted for an alternative approach to dichotomize tumor samples into high- and low-avidity cases. This was based on radioactivity measurements in tumor tissues derived from patients who received preoperative tracer doses of radioiodine.

## Materials and Methods

### Study Population and Radioactivity Handling

The study was conducted on a set of unique surgical specimens, with corresponding data on iodine concentration in tumor tissue, providing high-quality information on iodine avidity. Following approval from the Swedish Ethical Review Authority (#2020–01222, #2020–01541, and #2015_959-31), we selected a cohort of patients previously described [23]. This cohort consisted of adult patients with cytologically confirmed PTC and poorly differentiated thyroid carcinoma (PDTC) larger than 1 cm, all referred to Karolinska University Hospital, Stockholm, Sweden. Exclusion criteria included pregnancy, severe renal impairment (eGFR < 30 mL/min/1.7 m^2^), and cases where the primary tumor was too small for adequate tissue sampling. Tissue specimens from 27 patients, collected between 2019 and 2021, were selected for analysis. None of the patients had previously received radioiodine therapy. All the tumor samples were diagnosed according to the 2022 WHO classification of endocrine tumors, including main growth pattern, PTC subtyping, and also assessment of mitotic index and tumor necrosis to allow diagnosis of differentiated high-grade thyroid carcinoma (DHGTC) and PDTC. The molecular characterization was focused on detecting *BRAF* p.V600E mutants by immunohistochemistry, direct sequencing, and *TERT* promoter mutations (C228T and C250T) using digital droplet PCR.

The methodology of sample and radioactivity handling has been described in detail in a previous publication, studying other aspects of iodine avidity in a part of this cohort [24]. Briefly, two days preceding surgery, patients were administered a low-activity tracer injection of ^131^I (5–10 MBq). The low amount of radioactivity and the short interval between injection and surgery were chosen to ensure that the absorbed dose to tumor tissue remained well below the threshold known to affect iodine uptake or *NIS* mRNA expression (i.e., < 0.1 Gy) [25]. Following surgery, dissection of representative portions of the tumor and lymph node metastases (if present) was performed. In instances of multifocal primary tumor growth, the largest lesion was dissected. The radioactivity in tumor tissues was then quantified as normalized activity concentration (fraction of injected activity per gram of tissue: IA g^−1^) through measurements conducted in a gamma-counting scintillation detector. After radioactivity measurements, samples were fixed in formalin and embedded in paraffin (FFPE). The method of evaluating iodine avidity on surgical specimens has previously been shown to be predictive of subsequent metastatic uptake on nuclear imaging [26].

### Whole-Transcriptome Sequencing and Real-Time Reverse Transcription Polymerase Chain Reaction

Whole-transcriptome analysis (RNA-seq) was performed using 300 ng of total RNA from FFPE samples as described previously [27]. RNA with DV200 percentages ranging from 20 to 60%, underwent ribosomal RNA depletion and library preparation using the Illumina TruSeq Stranded Total RNA Library Preparation Kit (Illumina, Inc). Library preparation, sequencing, and gene expression quantification were conducted at the Science for Life Laboratory (SciLifeLab, Uppsala, Sweden). FastQC and MultiQC reports based on post-trimming data, were examined to assess the overall quality of sequencing data. Samples with poor quality scores, abnormal GC content distribution, or a high prevalence of overrepresented sequences were filtered out. The filtered high-quality reads were aligned to the human genome (GRCh-hg38) and analyzed with DESeq2 R.

A total of 700 ng of RNA per sample was reverse transcribed using a High-Capacity cDNA Reverse Transcription Kit with RNase Inhibitor (Applied Biosystems). RT-qPCR was then performed on the QuantStudio1 Real-Time PCR System (Applied Biosystem) using QuantiTect SYBR Green PCR Kit (Qiagen), following the manufacturer’s protocol. *GAPDH* served as endogenous control, and relative mRNA expression levels were calculated using the 2^−ΔΔCt^ method. All the primers are listed in Supplementary Table 1.

### Differential Expression Analysis and Functional Enrichment Analysis

Before differential expression analysis, transcripts were filtered based on the following criteria: (a) transcripts with a transcript per million (TPM)-normalized read count average across all samples < 6; (b) transcripts with a TPM value of 0 in more than 50% of samples; (c) transcripts associated with ribosomal, mitochondrial, and Golgi apparatus functions were excluded [28, 29]. The remaining 8833 transcripts were processed using the DESeq2 R package [30], with RAI avidity specified as the experimental design formula. DESeq2 normalized raw count data by calculating sample-specific size factors while testing for significant differences in gene expression between sample groups. Differential expression was assessed using negative binomial generalized linear models, with the Benjamini–Hochberg procedure applied to control the false discovery rate (FDR) in multiple testing. Differentially expressed genes (DEGs) were identified and reported with their associated Log_2_ fold changes (Log_2_FC).

Supervised analysis of DEGs was performed using principal component analysis (PCA). A volcano plot was generated to visualize the distribution of FDR against log2FC values. Hierarchical clustering of DEGs was performed on Log_2_-transformed read counts, and the resulting heatmap was used to visualize gene expression patterns across samples. DEGs were also analyzed for gene ontology (GO) and pathway enrichment using clusterProfiler and ReactomePA R packages, respectively.

### Immunohistochemistry

To validate the findings from our initial transcriptome analysis, immunohistochemistry (IHC) was conducted. This approach was employed to investigate and confirm specific protein expression levels in both the high-avidity and low-avidity groups. The IHC was performed on a Ventana BenchMark Ultra platform using a clinical routine protocol. Thus, FFPE tissues from the cohort were sectioned into 4-µm-thick slices and deparaffinized in xylene, followed by rehydration through a series of graded ethanol. Antigen retrieval was performed for 64 min using Ultra CC1, followed by the application of the ANO1/DOG1 antibody (clone SP31, 0.9 µg/mL, rabbit monoclonal, Roche Diagnostics, Basel, Switzerland) at room temperature for 32 min. Development was performed using UltraView DAB.

Immunoreactivity was assessed by an endocrine pathologist (CCJ) using light microscopy. For each slide, external controls were included in the form of a punch biopsy, consisting of a single de-identified gastrointestinal stromal tumor (GIST) as a positive control and normal colon and kidney tissues as negative controls. These controls ensured appropriate sensitivity and specificity for each staining reaction. Staining was categorized as negative (complete absence of ANO1 immunoreactivity), weak (faint positivity), moderate (average staining intensity), or strong (overtly positive staining). An H-score was calculated using the formula: 1 × percentage of weak staining + 2 × percentage of moderate staining + 3 × percentage of strong staining, resulting in a final score ranging from 0 to 300. Since ANO1 functions as an apical membrane transporter in thyroid follicular cells, we anticipated significantly weaker and less diffuse immunoreactivity compared to GIST lesions. Scoring was therefore conducted at × 400 magnification, with particular attention given to the apical membrane and cytoplasm. Nuclear expression was not expected.

### Statistical Analysis

The distribution of avidity and expression data was assessed. Normality was ascertained through histogram and Q-Q plot analysis. When required, data transformations were performed to achieve normality before further statistical analysis. Fisher’s exact test and the Mann–Whitney *U*-test were used to compare clinicopathological features between groups of high and low iodine avidity. Differential gene expression analysis was carried out using the *DESeq2* R package with the Wald test serving as the default method to identify DEGs. For this analysis, *p*-values were adjusted through the Benjamini–Hochberg procedure, and an FDR < 0.05 was considered statistically significant. The Mann–Whitney *U*-test was also used to assess differences in mRNA levels of selected genes between cases with low and high avidity cases. Linear correlations between iodine avidity and gene expression were calculated using Pearson’s product-moment correlation (*cor.test* in R).

Multivariate modelling was based on a previously established model and associated parameters, which in its best-performing form included histological subtype, TPO, NIS and Tg immunohistochemical expression, and *TERT* promoter mutation [23]. The model was expanded with all DEGs, in order to find the best independent predictors of avidity. Through the elimination of variables that did not increase the adjusted *R*^2^, a final model was established using the *lm* function in R. Any model that had clearly non-normally distributed or heteroscedastic residuals was discarded. Collinearity was assessed using variance inflation factors (*VIF* function in R). Continuous variables were transformed by logarithm to improve modelling results when beneficial.

Statistical analyses were performed using RStudio (R version 4.3.3, R-project.org) and GraphPad Prism (version 10.2.3; GraphPad Software, San Diego, CA, USA). A *p*-value < 0.05 was considered statistically significant for all direct comparisons.

## Results

### Clinicopathologic and Molecular Characteristics of the Study Population

A total of 36 FFPE sample cases from 27 patients, including 24 primary tumors and 12 lymph node metastases, were selected for the analysis. Ex vivo measurements revealed a broad range of iodine concentrations, varying over three orders of magnitude from the lowest detectable avidity value of 2 × 10^−6^ to 2 × 10^−3^ IA g^−1^. Iodine avidity was assessed and analyzed primarily as a continuous variable. However, for descriptive statistics and some analysis, two equally sized groups were created, consisting of low-avidity and high-avidity tumors. The groups were created by splitting samples at the median value of iodine concentration, which was 7 × 10^−5^). As earlier published analysis has shown, healthy thyroid tissue in the cohort had avidity values above 7 × 10^−3^ IA g^−1^, with a median value of 2 × 10^−2^ IA g^−1^ [24].

**Fig. 1:**
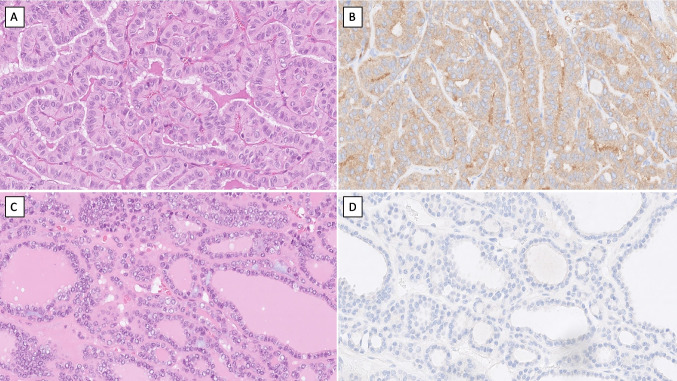
Histology of high- and low-avidity papillary thyroid carcinoma. **A** Hematoxylin and eosin (H&E) stained tall cell subtype of papillary thyroid carcinoma with *BRAF* p.V600E and C228T *TERT* promoter mutations. This tumor displayed low radioiodine avidity as measured ex vivo. **B** Clinical routine BRAF VE1 immunohistochemistry with a positive cytoplasmic signal in the same case. **C** H&E stained minimally invasive encapsulated follicular variant papillary thyroid carcinoma with high radioiodine avidity. **D** As expected, no BRAF VE1 immunoreactivity was noted. All images are magnified × 400

The clinical, histological, and molecular characteristics of each group are outlined in Table 1. Cases in the high-avidity group were generally younger at diagnosis, with an average age of 48.6 ± 13.8 compared to 59.3 ± 14.5 in the low-avidity group (*p* = 0.04). Primary tumors were more prevalent than metastases in the high-avidity group (15/18 vs. 9/18), although this difference was not statistically significant. While the majority of PTCs with unfavorable histology (9/18; 50%) were low-avidity cases (Fig. 1A, B), and the same proportion of cases with favorable histology were high-avidity cases (9/18; 50%) (Fig. 1C, D), this trend did not reach statistical significance. Regarding high-grade thyroid carcinoma, one case met the criteria for DHGTC, and one case fulfilled the diagnostic criteria for PDTC (Table 1). There was no progression in terms of tumor grade noted in any of the paired cases (primary tumor and lymph node metastases). No significant differences were noted in sex distribution, primary tumor size, or metastasis size.

**Table 1:** Clinical, histological, and molecular characteristics of samples with RAI avidity measurement

Clinical features	High avidity	Low avidity	All samples	*p*-value
	*n* = 18	*n* = 18	*n* = 36	
Age (years)				0.04*
Mean ± SD	48.6 ± 13.8	59.3 ± 14.5	53.9 ± 15.0	
Median (range)	50.5 (19–81)	56 (26–79)	53 (19–81)	
Sex				0.73
Female	10 (55.6%)	9 (50%)	19 (52.8%)	
Male	8 (44.4%)	9 (50%)	17 (47.2%)	
Tumor type				0.07
Primary tumor	15 (83.3%)	9 (50%)	24 (66.7%)	
Metastasis	3 (16.7%)	9 (50%)	12 (33.3%)	
Tumor size (mm)				
Primary tumor				
Mean ± SD	30.3 ± 16.4	29.9 ± 4.4	30.1 ± 13.3	0.86
Median (range)	27 (14–70)	30 (21–35)	29 (14–70)	
Metastasis				
Mean ± SD	24 ± 3.6	20.7 ± 14.1	21.5 ± 12.2	0.31
Median (range)	25 (20–27)	16 (1–40)	22.5 (1–40)	
pT				-
pT1a	0 (0%)	2 (11.1%)	2 (5.6%)	
pT1b	7 (38.9%)	1 (5.6%)	8 (22.2%)	
pT2	6 (33.3%)	12 (66.7%)	18 (50.0%)	
pT3a	2 (11.1%)	1 (5.6%)	3 (8.3%)	
pT3b	3 (16.7%)	2 (11.1%)	5 (13.9%)	
pN				
pN0	1 (5.6%)	3 (16.7%)	4 (11.1%)	
pNx	7 (38.9%)	2 (11.1%)	9 (25.0%)	
pN1a	5 (27.8%)	3 (16.7%)	8 (22.2%)	
pN1b	5 (27.8%)	10 (55.6%)	15 (41.7%)	
Histology				-
PTC *favorable*	9 (50%)	7 (38.9%)	16 (44.4%)	
CPTC	6	6	12	
FVPTC	2	-	2	
WLPTC	1	1	2	
PTC *unfavorable*	7 (38.9%)	9 (50%)	16 (44.4%)	
DSPTC	3	2	5	
TCPTC	3	6	9	
OPTC	1	1	2	
DHGTC	-	1 (5.6%)	1 (2.8%)	
PDTC	2 (11.1%)	1 (5.6%)	3 (8.3%)	
Mutational status				*_*
WT	7 (38.9%)	5 (27.8%)	12 (33.3%)	
*BRAF* p.V600E	8 (44.4%)	4 (22.2%)	12 (33.3%)	
*BRAF* p.V600E + *TERT* C228T/C250T	3 (16.7%)	7 (38.9%)	5 (27.8%)	
NA	-	2 (11.1%)	2 (5.6%)	

*RAI* radioiodine, *PTC* papillary thyroid carcinoma, *CPTC* classic PTC, *FVPTC* follicular variant PTC, *WLPTC* Warthin-like PTC, *DSPTC* diffuse sclerosing PTC, *TCPTC* tall cell PTC, *OPTC* oncocytic PTC, *DHGTC* differentiated high-grade thyroid carcinoma, *PDTC* poorly differentiated thyroid carcinoma, *NA* not available, *SD* standard deviation, *WT* wild-type

^*^Statistically significant

As previously reported [23], the mutational analysis of the cohort revealed a high prevalence of the *BRAF* p.V600E mutation, detected in 22 out of 36 cases. Among the high-avidity tumors, 11 cases (61%) harbored the *BRAF* p.V600E mutation, with 3 (27%) of these also carrying the *TERT* promoter mutation. Similarly, in the low-avidity group, 11 cases (61%) exhibited the *BRAF* p.V600E mutation, and among these, 7 (64%), carried the *TERT* promoter mutation. Twelve cases were wild-type for both *BRAF* and *TERT* mutations (7/18 in high-avidity vs. 5/18 in low-avidity). Two low-avidity cases failed the mutational analysis. The failed DNA analysis was likely due to the long storage time of the paraffin blocks, which may affect DNA quality and yield.

Clinical and nuclear imaging correlates in our cohort have been previously published [26]. Briefly, data from 35 patients showed that 10 developed or had persistent disease during follow-up (range: 19–46 months). Among these, four patients had non-avid persistent metastatic disease, all of whom had low iodine avidity in their primary tumors and initial lymph node metastases. The results confirmed that pre-therapeutic ex vivo measurements used in this work agree with uptake on subsequent nuclear imaging of metastases.

### Transcriptomic Characterization Associated with Iodine Avidity

**Fig. 2:**
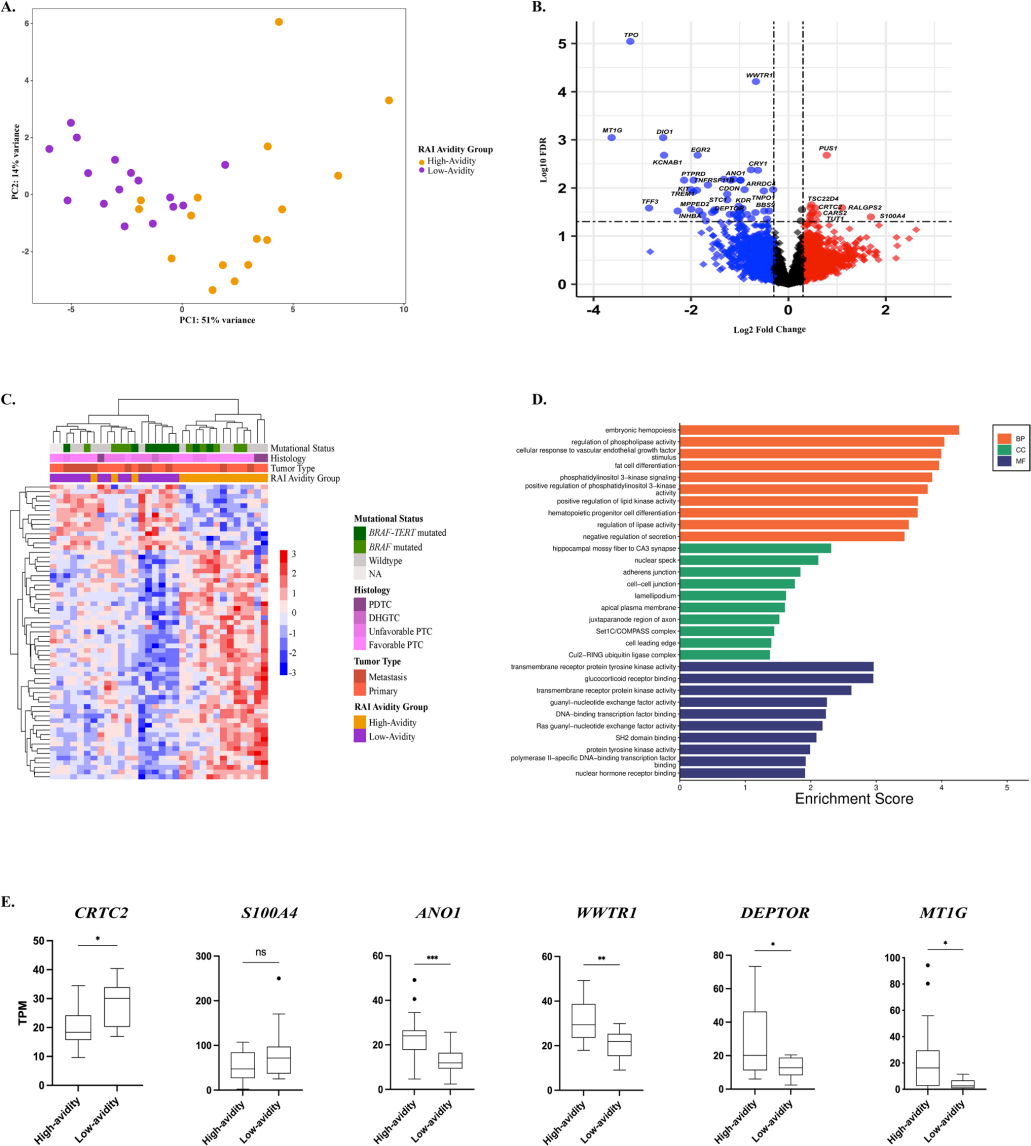
Gene expression analysis of high-avidity and low-avidity cases. **A** Principal component analysis (PCA) plot based on high-avidity (*n* = 16) and low-avidity (*n* = 16) tumors. Each dot corresponds to an individual tumor case. **B** Volcano plot showing the most dysregulated genes between high- and low-avidity tumor cases. **C** Heatmap of the 63 differentially expressed genes (DEGs) between the two groups of cases analyzed. All the low-avidity cases are clustered together on the left part of the graph, while the high-avidity cases are clustered on the right. Three high-avidity cases were recognized as low-avidity. **D** Bar plot of representative gene ontology (GO) terms categorized into three main functional groups: biological process (BP) in orange, cellular component (CC) in green, and molecular function (MF) in blue. Within each group, the top ten significative (*p*-value < 0.05) GO terms are displayed on the y-axis and ranked by their enrichment score (x-axis), with higher scores indicating greater enrichment significance. **E** Expression level of the most consistent deregulated genes between high- and low-avidity cases detected by RNA-seq. *CRTC2* and *S100A4* were found to be upregulated in low-avidity cases compared with high-avidity, while *ANO1*, *WWTR1*, *DEPTOR*, and *MT1G* were downregulated in low-avidity cases compared with high-avidity

RNA-seq data derived from 36 cases were analyzed for differential gene expression. Four cases were ruled out due to low-quality RNA, as highlighted in the MultiQC report. To identify differences in gene expression profiles and detect specific genes associated with the refractory tumor phenotype, we conducted a supervised analysis of RNA-seq data from 16 tumors in the high-avidity group and 16 in the low-avidity group. Whole-transcriptome analysis showed distinct clustering of high-avidity and low-avidity tumors (Fig. 2A). Differential expression analysis identified 63 genes that were significantly differentially expressed between the two groups (FDR < 0.05). Of these, 14 genes were upregulated, while 49 genes were downregulated in low-avidity tumors compared to high-avidity tumors. Among the 63 DEGs, we identified six that were consistently deregulated and biologically relevant to tumorigenesis and thyroid metabolism, potentially influencing the regulation of RAI avidity in thyroid cancer (Fig. 2B).

The heatmap in Fig. 2C shows the differential gene expression profile for each group. The first cluster (on the left) consisted predominantly of low-avidity phenotype cases. Interestingly, three cases, of which one oncocytic PTC (OPTC) and two tall cell PTCs (TCPTC), deviated from the high-avidity cluster and instead grouped with the low-avidity cases. Upon further investigation, the OPTC case exhibited a borderline avidity value, while one of the TCPTC cases developed distant metastases with low avidity during clinical follow-up observations. The second main cluster (on the right) grouped the remainder of the high-avidity phenotype cases, a distinct subgroup of two PDTC cases positioned at the far right. One of these cases exhibited distinct gene expression patterns compared to the low-avidity cases, and clinical follow-up indicated that the corresponding patient developed distant metastases with high RAI avidity.

From the 63 DEGs, we next identified six that were consistently deregulated and have known biological relevance to tumorigenesis and thyroid metabolism, likely impacting the regulation of RAI avidity in thyroid cancer. Among these, two genes (*S100A4* and *CRTC2*) were found to be upregulated, while four genes (*ANO1*, *WWTR1*, *DEPTOR*, and *MT1G*) were downregulated in low-avidity compared to high-avidity cases, as determined by TPM values (Fig. 2E).

**Fig. 3:**
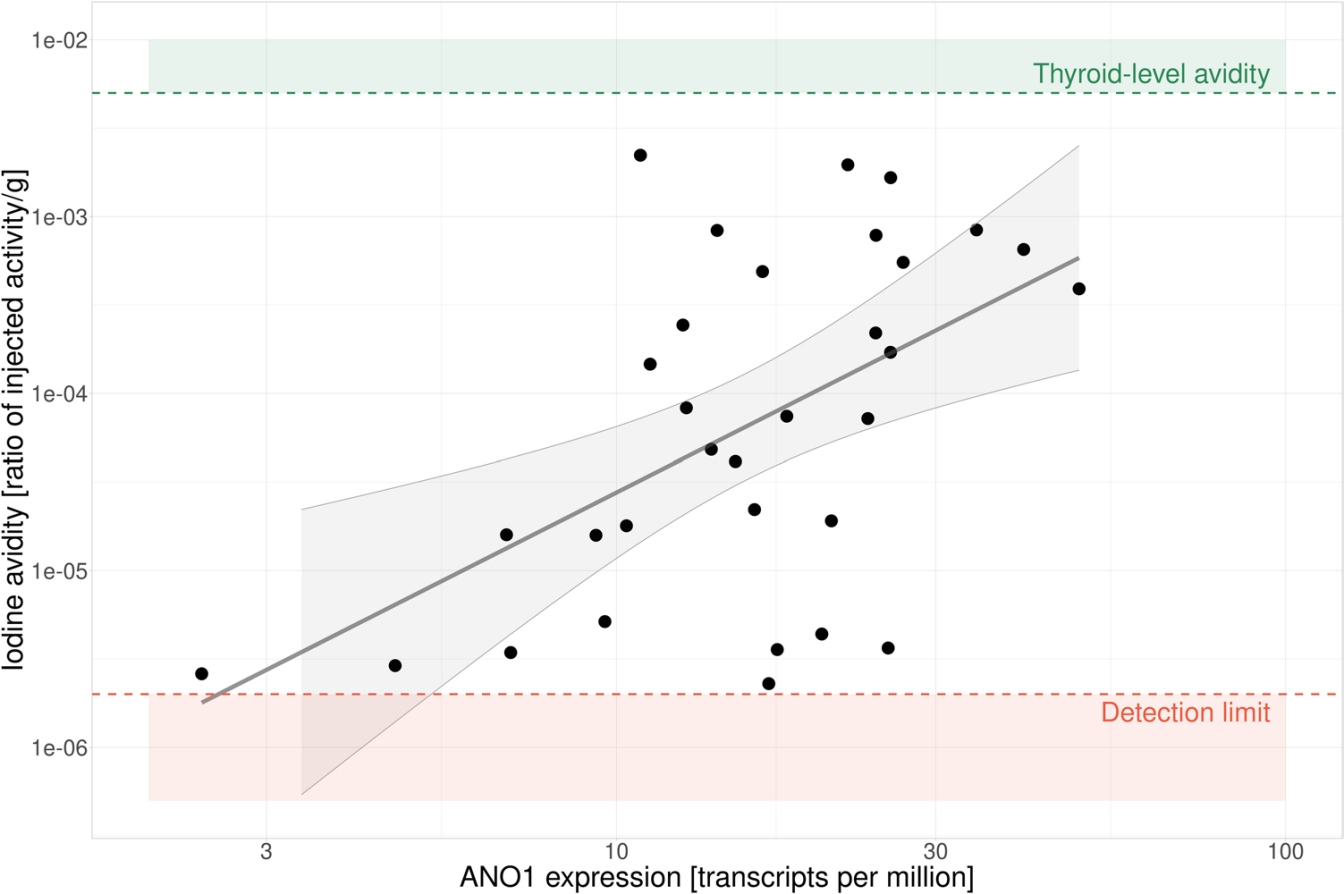
Correlation between *ANO1* mRNA expression and iodine avidity in tumor tissue. The correlation coefficient was *r* = 0.54, CI 0.23–0.75, *p* < 0.01, with 83-fold higher avidity for a tumor with tenfold higher *ANO1* expression. Levels of avidity observed in normal thyroid tissue (green), the lower detection limit for avidity in the experiment (orange), and the linear correlation line with confidence intervals (grey) are also displayed for clarity

Furthermore, the expression of the six DEGs was tested for correlation with iodine avidity as a continuous variable. As reported in Fig. 3, the results showed a significant positive correlation between avidity and *ANO1* expression (*r* = 0.54, CI 0.23–0.75). A 83-fold higher tumor avidity for iodine was observed for a tenfold higher *ANO1* expression in the tissue. No other gene expression among the six DEGs showed statistically significant correlations with avidity.

To further validate these findings, RT-qPCR was then performed on a subset of cases with still available RNA or tissue (8 low-avidity and 8 high-avidity). Although the upregulation of *S100A4* in low-avidity tumors was not statistically significant (*p* = 0.12), this gene was included in the validation list of genes due to its established role in thyroid cancer and its involvement in tumorigenesis across various other cancer types (Supplementary Fig. 1). While the differences in mRNA expression levels of *ANO1* and *WWTR1* between low- and high-avidity cases were not statistically significant, both genes exhibited the same trend of lower expression in low-avidity tumors. Conversely, *CRTC2* and *S100A4* mRNA levels were higher in low-avidity tumors, with statistical significance observed for *S100A4*, corroborating the findings from the RNA-seq analysis.

In addition to the genes that showed consistent dysregulation, *SLC5A5* (encoding NIS) was investigated separately due to its known association with thyroid cancer and iodine metabolism. Excluding those samples with TPM = 0, the analysis showed a significant positive correlation between *SLC5A5* expression and iodine avidity (*r* = 0.38, CI 0.01–0.67) (Supplementary Fig. 4). Similarly, comparing the high- and low-avidity groups, high-avidity samples exhibited a 5.8-fold higher expression (CI 1.5–23).

### Gene Ontology and Pathway Enrichment Analysis

To explore the biological significance of the previously identified DEGs, gene ontology (GO) and pathway enrichment analyses were conducted. GO analysis classified the DEGs into functional categories, including biological processes (BP), molecular functions (MF), and cellular components (CC). Figure 2D highlights the top 10 significantly enriched BP, MF, and CC categories, ranked according to their enrichment scores. Although not shown in Fig. 1D, the GO analysis revealed significant enrichment in processes specifically related to the thyroid, such as “thyroid hormone generation” and “thyroid hormone metabolic processes.” Additionally, several biological processes and molecular functions associated with cell adhesions and wound healing, including “response to vascular endothelial growth factor stimulus,” “adherens junction,” and “cell–cell junction,” were also significantly enriched. These processes have been previously linked to more advanced stages of thyroid cancer [21]. Notably, the enrichment of “phosphatidylinositol 3-kinase signaling” aligns with the role of this pathway in regulating RAI avidity. Moreover, pathway enrichment analysis (Supplementary Fig. 2) highlighted significant enrichment in the PI3K-AKT and MAPK signaling pathways, as well as pathways involved in thyroid hormone synthesis, underscoring the crucial role these processes play in influencing iodine avidity.

### ANO1 Immunohistochemistry

**Fig. 4:**
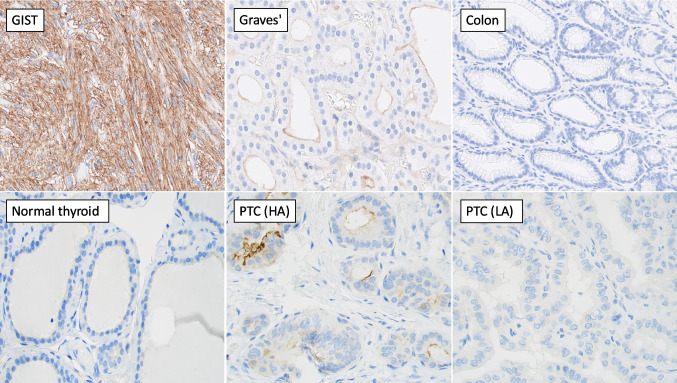
ANO1 immunoreactivity in papillary thyroid carcinoma (PTC) with variations in radioiodine avidity. All photomicrographs were captured at × 400 magnification. The top row shows control experiments for each slide, demonstrating strong cytosolic ANO1 expression in a gastrointestinal stromal tumor (GIST) and weaker membranous staining in a de-identified case of Graves’ diffuse hyperplasia as well as negative immunoreactivity in normal colon. The bottom row illustrates weak and focal ANO1 expression in adjacent normal thyroid tissue followed by two PTC cases, one with high iodine avidity (HA) and one with low (LA). The H scores shown are 150 for the avid case and 0 for the non-avid case. Note that in the thyroid samples, ANO1 expression is localized to the apical membrane

ANO1 immunoreactivity is illustrated in Fig. 4, along with controls. Overall, the immunoreactivity in thyroid tumors was weak and variable, also in adjacent normal thyroid tissues. Notably, ANO1 expression was confined to the apical membrane and was never detected in the cytosol or nuclear compartment. H-scores ranged from 0 to 150 (data not shown), with a median value of 25 indicating faint expression localized to the apical membrane.

The correlation coefficient between ANO1 immunohistochemical H-score and iodine avidity was *r* = 0.51 (CI 0.07–0.78, *p* = 0.03) (Supplementary Fig. 3). However, the correlation between ANO1 H-score and mRNA expression was not significant, with *r* = 0.28 (CI − 0.23–0.67, *p* = 0.27).

### Multivariate Modelling

The multivariate modelling used the DEGs found in the RNA-seq (*ANO1, WWTR1, S100A4, CRTC2, GLIS3, *and* NKX2_1*). Additionally, the expression of *SLC5A5* was tested in the modelling due to its well-established role in iodine transport through the plasma membrane. The process of elimination of variables resulted in a predictive model with *ANO1* expression performing well. Markers from the previously established model that still performed well were immunohistochemical Tg expression, high-risk histology (tall cell and hobnail variant PTC, DHGTC, and PDTC), and type of sample (primary tumor vs. lymph node metastasis). The resulting model was less complex than previously described while performing similarly in terms of predictive power (adjusted *R*^2^ of 0.55). Therefore, data on ANO1 expression likely improve any predictive scheme for iodine avidity. Replacing *ANO1* mRNA expression with ANO1 intensity score from immunohistochemistry resulted in a similar, albeit slightly lower, model performance (adjusted *R*^2^ of 0.48). While the modelling results were used to select the most impactful predictors of iodine avidity, no external validation was included, and the results are therefore not suited for generalization.

## Discussion

The rising incidence of thyroid cancer has become a significant public health concern, with an increasing number of patients undergoing surgical intervention and subsequent RAI therapy. While PTC generally carries an excellent prognosis, overtreatment remains a pressing issue, particularly given that most patients experience favorable outcomes without aggressive therapeutic interventions. Consequently, there is a growing emphasis on the de-escalation of thyroid cancer treatment, aiming to balance therapeutic efficacy with the minimization of unnecessary adverse effects.

RAI therapy remains a cornerstone in the management of differentiated thyroid cancer; however, its efficacy is contingent on sufficient iodine avidity of the tumor cells. Tumors that fail to concentrate iodine exhibit resistance to RAI therapy, limiting treatment options and contributing to poorer clinical outcomes. Therefore, early identification of cases likely to be or become RAI-R is of crucial importance. A key challenge lies in distinguishing these refractory cases at an early stage, ideally before initial RAI administration, to better guide individualized therapeutic strategies. Our study presents a novel approach by analyzing global RNA expression in surgical specimens using ex vivo iodine concentration measurements. This methodology sets our cohort apart, as previous studies have predominantly relied on clinical scintigraphy performed after RAI administration, which assesses iodine avidity uptake with poor spatial resolution in vivo. Such approaches are practical if RAI imaging is included in the clinical routine but may fail to capture the contemporary, intrinsic biological, and molecular characteristics influencing iodine avidity. This potentially overlooks critical predictive biomarkers that otherwise may change in expression during the course of treatment. Interestingly, given that the observed iodine avidity ex vivo varied across several orders of magnitude, many associations were evident despite the small sample size, indicating that the method is much more sensitive than post-therapeutic imaging.

Through transcriptomic analysis, we identified 63 DEGs between high- and low-avidity tumors, with six genes (*S100A4*, *CRTC2*, *ANO1*, *WWTR1*, *DEPTOR*, *MT1G*) showing consistent deregulation. Notably, the calcium-activated anion channel ANO1, located on the apical membrane of thyroid follicular cells and known to facilitate iodide efflux into the follicular lumen [31], displayed a significant correlation with iodine avidity (*r* = 0.54). The strong association between ANO1 expression and iodine avidity underscores its potential as a robust biomarker for predicting RAI responsiveness. *ANO1* is also known as *DOG1*, a marker of gastrointestinal stromal tumors. However, only single studies have analyzed ANO1/DOG1 immunoreactivity in thyroid tumors [32]. Interestingly, functional studies have shown that ANO1 may be responsible for most of the iodide efflux across the apical membrane of thyroid cells [31, 33, 34]. Therefore, the finding of *ANO1* downregulation in thyroid cancers with low iodine avidity is plausible and may suggest that this gene constitutes a potentially important differentiation marker in subsets of thyroid neoplasia.

Given the established role of *SLC5A5*/NIS in the thyroid, we performed selected analyses of this gene. The analysis of *SLC5A5* expression in this study revealed a significant correlation with iodine avidity both as a continuous variable and in the two avidity groups, where *SLC5A5* mRNA levels were 5.8-fold higher in and high- versus low-avid cases. Previous analysis of NIS IHC expression in the same cohort showed that only a small number of tumors exhibited clear membranous localization of NIS, and these cases were associated with dramatically higher iodine avidity [23]. In contrast, many iodine-avid tumors displayed low or absent NIS expression on immunohistochemistry, which may be indicative of the low endogenous levels of this protein in thyroid tissues. These findings suggest that immunohistochemistry alone may not be sufficient to fully capture the relationship between NIS expression and iodine avidity in thyroid cancer.

Furthermore, pathway enrichment analyses of our cohort highlighted critical molecular pathways, including thyroid hormone synthesis, PI3K-AKT, and MAPK signaling, which may play pivotal roles in modulating iodine uptake. These findings not only enhance our understanding of the molecular mechanisms underlying RAI refractoriness but also open avenues for targeted therapeutic interventions aimed at restoring or enhancing iodine avidity in refractory tumors. Previous studies using mRNA analyses in clinically aggressive PTCs have identified various pathways that are differentially regulated in non-aggressive compared to aggressive PTC, including the DNA damage repair, MAPK, and RAS pathways, which partly align with our findings [35].

Our predictive model, which integrates *ANO1* mRNA expression, histological subtype, and sample type, demonstrated strong predictive performance (adjusted *R*^2^ = 0.55). While the model is not validated on a separate cohort, limiting conclusions to indications, it served as a tool to find the most impactful predictor of avidity. The modelling results showed the potential impact of *ANO1* expression on iodine avidity, outperforming several other prominent markers. ANO1 immunoreactivity as a marker performed slightly worse than mRNA expression in prediction modelling, but the results may still hint at a role for a simpler work-up. Moreover, ANO1 immunohistochemistry may not be a practical substitute for detecting cases with reduced ANO1 expression. The expression was both variable and weak and challenging to detect, as it was confined to the apical membrane. For any marker to be useful in clinical practice, it must offer a clear interpretation. Therefore, we are not in a position to recommend ANO1 screening for routine clinical practice. Additionally, the correlation between immunoreactivity and iodine avidity was not as strong as that observed for mRNA expression analyses. Although mRNA analyses may pose a challenge for many laboratories in evaluating this marker, we believe that alternative methods, such as in situ hybridization may offer better options for detecting ANO1 downregulation in clinical samples. This issue is similar to that observed with other iodine transporters in thyroid follicular cells, such as NIS, where immunohistochemical studies have struggled to find relevant correlations to mRNA expression, likely due to both low endogenous levels and a highly polar distribution within the cells. Nevertheless, the trend persists: the expression of various iodine transporters and other genes and corresponding proteins associated with thyroid hormone production, including thyroglobulin, reflects the differentiation status of the tumor cells. Therefore, it is plausible that ANO1 downregulation could contribute to the molecular mechanisms underlying thyroid cancer dedifferentiation.

Our study has limitations. First, we present a relatively small tumor cohort, which is not entirely surprising given the study protocol, which involved preoperative radioiodine administration to a selected group of patients. The cohort was further reduced, as not all resected samples were suitable for radioiodine uptake measurements, primarily due to small tumor sizes. This small sample size limits the ability to establish any less pronounced associations between PTC subtypes and radioiodine avidity. Additionally, the number of cases of DHGTC and PDTC is too low to allow for meaningful comparisons of RNA patterns. We also acknowledge the limitations of performing RNA sequencing on RNA extracted from FFPE tissues, as these samples are generally of lower quality compared to fresh-frozen samples. Furthermore, we note the lack of independent validation of *ANO1* expression. Lastly, since patients were enrolled continuously regardless of their genetic profiles, our cohort contains a disproportionate number of BRAF-like PTCs and few RAS-like tumors.

In conclusion, our study suggests that assessing iodine avidity directly from surgical specimens is a feasible approach and identifies *ANO1* as a potential biomarker for predicting RAI avidity in our cohort of PTCs, which is enriched for *BRAF*-driven tumors. This approach provides a potential advancement in the early identification of RAI-R thyroid cancers, possibly facilitating more personalized treatment strategies. Future studies should focus on validating these findings in larger cohorts and explore the therapeutic modulation of identified pathways to improve management and outcomes for patients with RAI-R disease.

## Supplementary Information

Below is the link to the electronic supplementary material.

Supplementary file1 Supplementary Figure 1. Validation analysis of the most consistent deregulated genes between high- and low-avidity cases. Expression level of 4 out of 6 genes using RT-qPCR. The Box plots clearly show the expression trend of the genes as shown in RNA-seq analysis. (TIF 739 KB)

Supplementary file2 Supplementary Figure 2. Pathway enrichment analysis of DEGs in low-avidity vs. high-avidity cases. Dotplot of representative GO terms enriched among DEGs. The color scale from red to blue indicates the ranges of significant p-value (< 0.05) and the diameter of each single point correlates with the number of genes counted. (TIF 561 KB)

Supplementary file3 Supplementary Figure 3. Correlation between ANO1 H-score detected by immunohistochemistry and iodine avidity in tumor tissue. The correlation coefficient was r=0.51 (CI 0.07 - 0.78, p=0.03). Levels of avidity observed in normal thyroid tissue (green), the lower detection limit for avidity in the experiment (orange), and the linear correlation line with confidence intervals (grey) are also displayed for clarity. (TIF 4185 KB)

Supplementary file4 Supplementary Figure 4. Correlation between SLC5A5 mRNA expression and iodine avidity in tumor tissue. The correlation coefficient was (r=0.38, CI 0.01-0.67), p<0.05. Levels of avidity observed in normal thyroid tissue (green), the lower detection limit for avidity in the experiment (orange), and the linear correlation line with confidence intervals (grey) are also displayed for clarity. (TIF 4621 KB)

Supplementary file5 Supplementary Table 1. List of primers used for RT-qPCR analysis. (XLSX 10 KB)

## Data Availability

No datasets were generated or analysed during the current study.
