# Supplementary figures and images for: Comprehensive Gene Expression Analysis in Papillary Thyroid Carcinoma Reveals a Transcriptional Profile Associated with Reduced Radioiodine Avidity
Source: Endocr Pathol. 2025 Feb 21;36(1):4. doi: 10.1007/s12022-025-09849-0 (PMC11845550; doi:10.1007/s12022-025-09849-0)

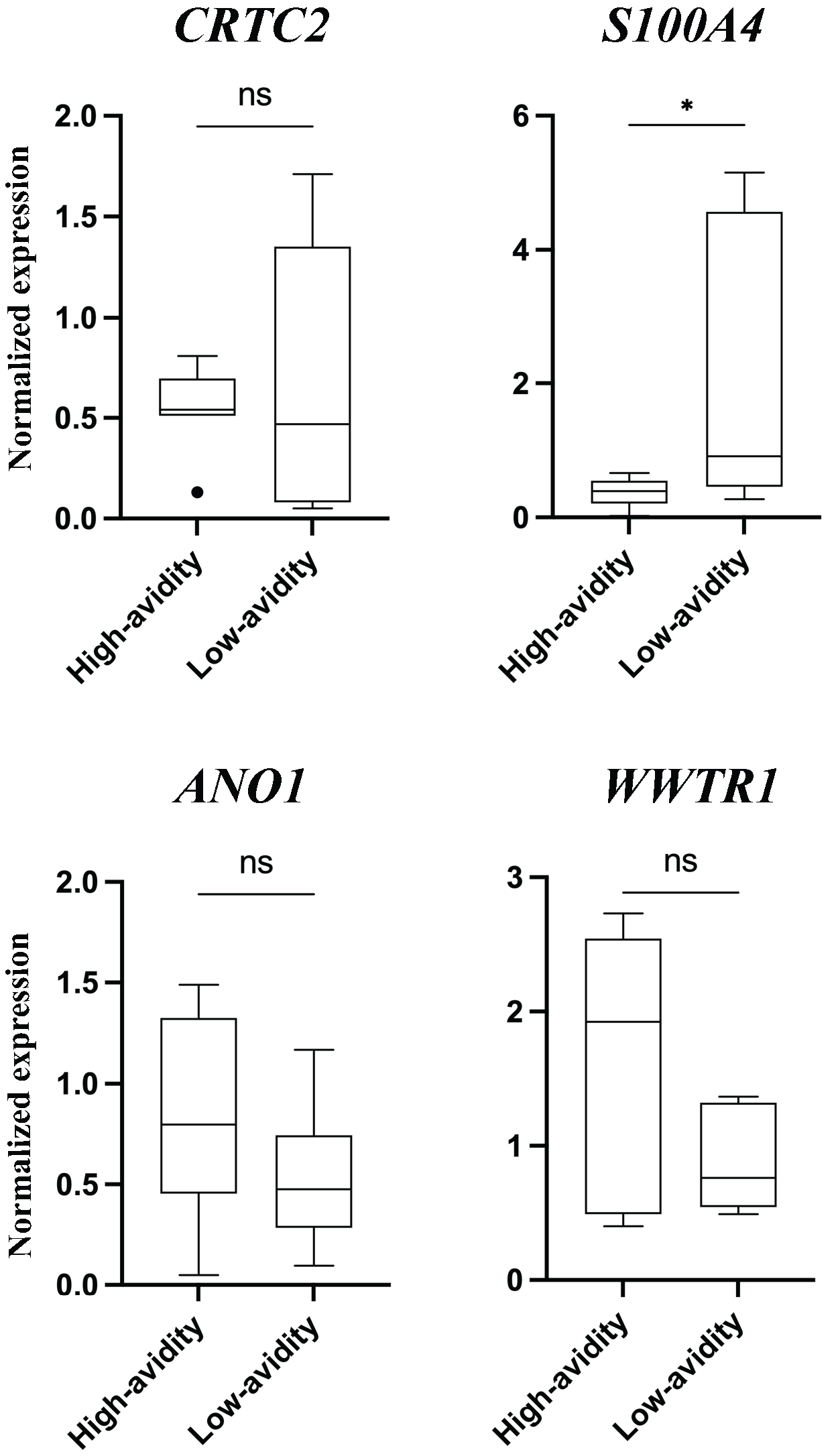

Supplement: Supplementary file 1 — Supplementary file1 Supplementary Figure 1. Validation analysis of the most consistent deregulated genes between high- and low-avidity cases. Expression level of 4 out of 6 genes using RT-qPCR. The Box plots clearly show the expression trend of the genes as shown in RNA-seq analysis. (TIF 739 KB) [file 12022_2025_9849_MOESM1_ESM.tif]

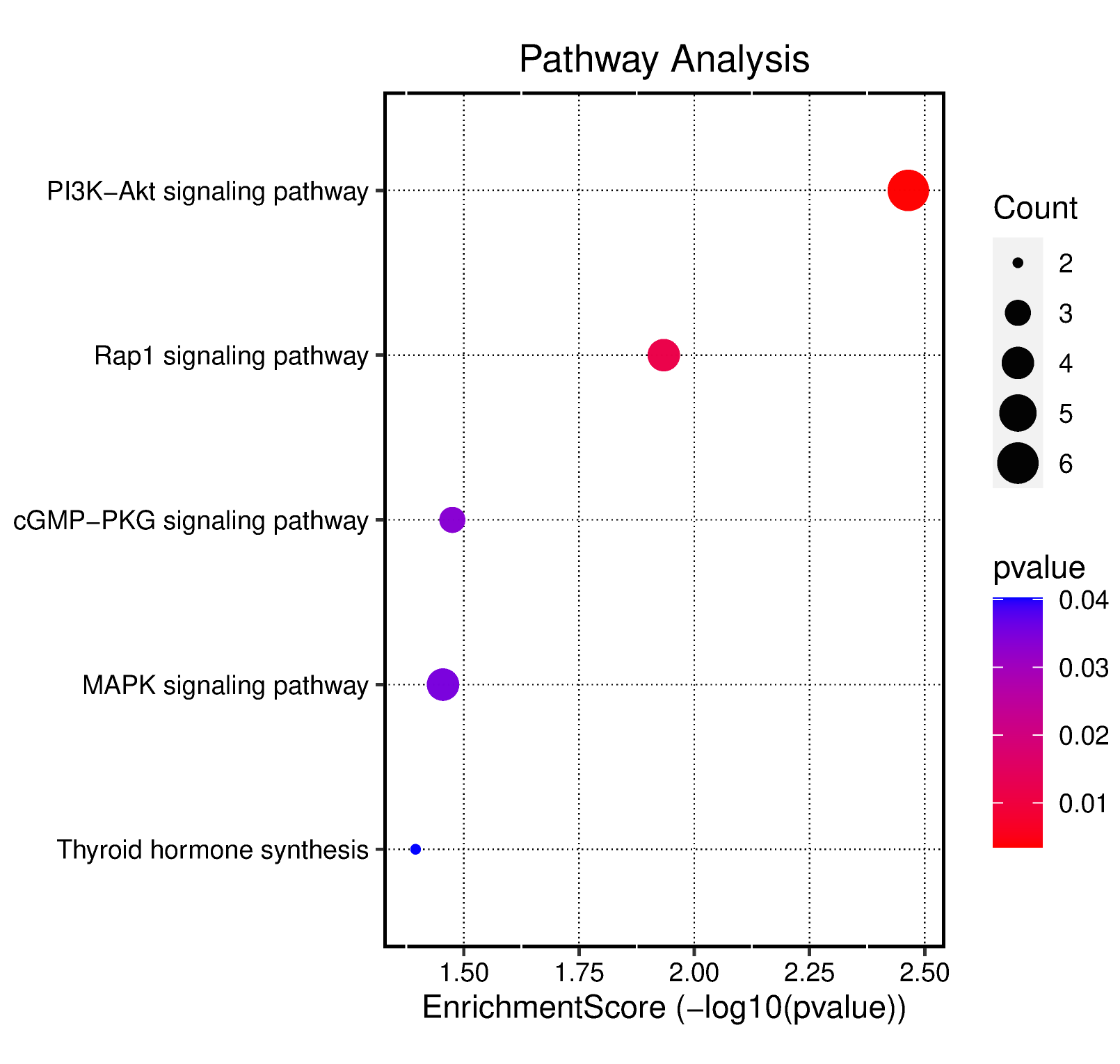

Supplement: Supplementary file 2 — Supplementary file2 Supplementary Figure 2. Pathway enrichment analysis of DEGs in low-avidity vs. high-avidity cases. Dotplot of representative GO terms enriched among DEGs. The color scale from red to blue indicates the ranges of significant p-value (< 0.05) and the diameter of each single point correlates with the number of genes counted. (TIF 561 KB) [file 12022_2025_9849_MOESM2_ESM.tif]

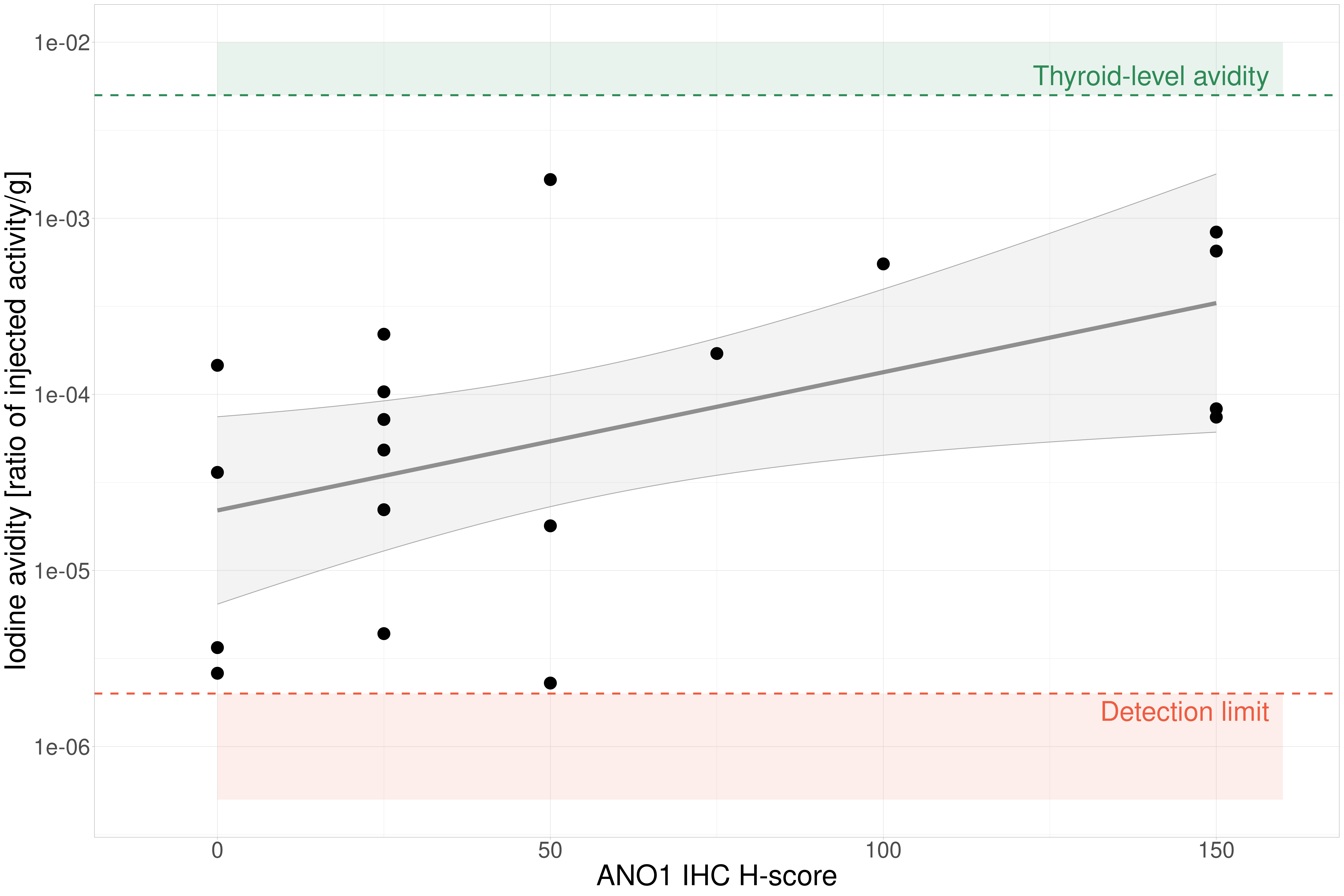

Supplement: Supplementary file 3 — Supplementary file3 Supplementary Figure 3. Correlation between ANO1 H-score detected by immunohistochemistry and iodine avidity in tumor tissue. The correlation coefficient was r=0.51 (CI 0.07 - 0.78, p=0.03). Levels of avidity observed in normal thyroid tissue (green), the lower detection limit for avidity in the experiment (orange), and the linear correlation line with confidence intervals (grey) are also displayed for clarity. (TIF 4185 KB) [file 12022_2025_9849_MOESM3_ESM.tif]

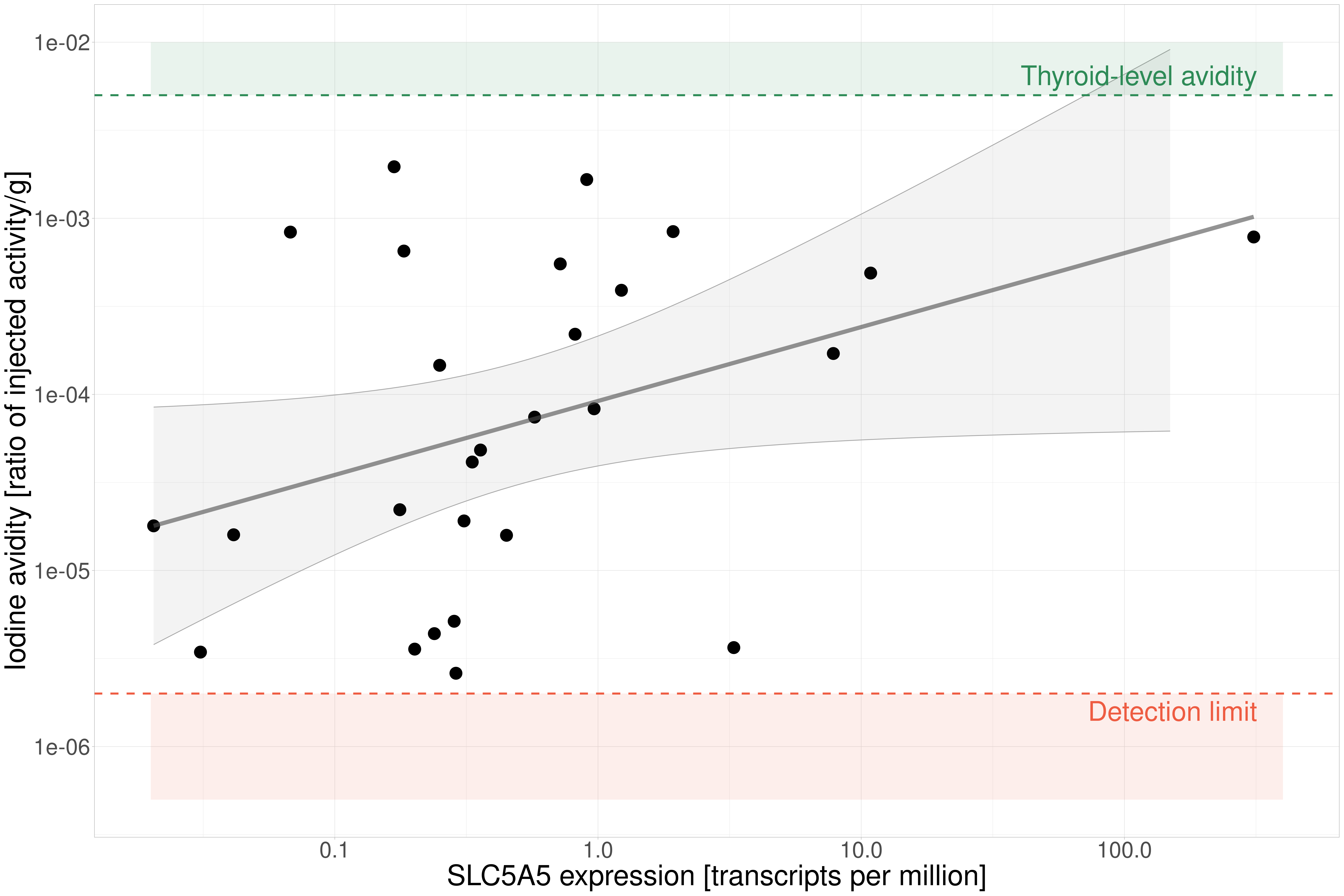

Supplement: Supplementary file 4 — Supplementary file4 Supplementary Figure 4. Correlation between SLC5A5 mRNA expression and iodine avidity in tumor tissue. The correlation coefficient was (r=0.38, CI 0.01-0.67), p<0.05. Levels of avidity observed in normal thyroid tissue (green), the lower detection limit for avidity in the experiment (orange), and the linear correlation line with confidence intervals (grey) are also displayed for clarity. (TIF 4621 KB) [file 12022_2025_9849_MOESM4_ESM.tif]
